# Microbial Source Tracking as a Method of Determination of Beach Sand Contamination

**DOI:** 10.3390/ijerph19137934

**Published:** 2022-06-28

**Authors:** Elisabete Valério, Maria Leonor Santos, Pedro Teixeira, Ricardo Matias, João Mendonça, Warish Ahmed, João Brandão

**Affiliations:** 1 Departamento de Saúde Ambiental, Instituto Nacional de Saúde Doutor Ricardo Jorge, Avenida Padre Cruz, 1649-016 Lisboa, Portugal; leonorsantos.22.66@gmail.com (M.L.S.); pedro.teixeira@cm-lisboa.pt (P.T.); joao.brandao@insa.min-saude.pt (J.B.); 2 Centre for Environmental and Marine Studies (CESAM), Department of Animal Biology, University of Lisboa, 1349-017 Lisboa, Portugal; 3 Câmara Municipal de Lisboa, Direção Municipal do Ambiente, Estrutura Verde, Clima e Energia, Laboratório de Bromatologia e Águas, Avenida Cidade do Porto S/N, 1700-111 Lisboa, Portugal; 4 Unidade de Desporto e Promoção da Qualidade de Vida, 9700-042 Angra do Heroísmo, Portugal; rmatias@cmah.pt; 5 Delegação de Saúde de Angra do Heroísmo, 9700-121 Angra do Heroísmo, Portugal; joaombmendonca@gmail.com; 6 CSIRO Land and Water, Ecosciences Precinct, 41 Boggo Road, Dutton Park 4102, Australia; warish.ahmed@csiro.au

**Keywords:** beach, coastal sand, fecal contamination, fecal indicator bacteria (FIB), microbial source tracking (MST)

## Abstract

Beach sand may act as a reservoir for numerous microorganisms, including enteric pathogens. Several of these pathogens originate in human or animal feces, which may pose a public health risk. In August 2019, high levels of fecal indicator bacteria (FIB) were detected in the sand of the Azorean beach Prainha, Terceira Island, Portugal. Remediation measures were promptly implemented, including sand removal and the spraying of chlorine to restore the sand quality. To determine the source of the fecal contamination, during the first campaign, supratidal sand samples were collected from several sites along the beach, followed by microbial source tracking (MST) analyses of *Bacteroides* marker genes for five animal species, including humans. Some of the sampling sites revealed the presence of marker genes from dogs, seagulls, and ruminants. Making use of the information on biological sources originating partially from dogs, the municipality enforced restrictive measures for dog-walking at the beach. Subsequent sampling campaigns detected low FIB contamination due to the mitigation and remediation measures that were undertaken. This is the first case study where the MST approach was used to determine the contamination sources in the supratidal sand of a coastal beach. Our results show that MST can be an essential tool to determine sources of fecal contamination in the sand. This study shows the importance of holistic management of beaches that should go beyond water quality monitoring for FIB, putting forth evidence for beach sand monitoring.

## 1. Introduction

Coastal areas provide a variety of recreational, athletic, and leisure activities, such as swimming, diving, water sports, and fishing [1]. However, many other activities do not require direct contact with water, as they take place on the sand or near the shoreline, such as running and walking along the seafront, practicing sand sports, children’s activities such as building sandcastles, socializing, and tanning [2,3,4,5,6,7,8]. The fascination with these coastal recreational environments tends to attract many users of all age groups and health conditions. This attraction ends up becoming an important local and often national economic revenue source.

Considering the relevance and frequent use of these spaces and recognizing that people spend more time on the sand than in water [1,8,9], it becomes evident that in addition to the need to maintain the quality of recreational waters, there is also a need to consider the quality of beach sands [10]. In a study conducted in Azores [11], the authors raised some awareness about this issue, showing that sand can be a public health threat. That study investigated the origin of a skin-rash outbreak caused by sodium hypochlorite. Following early cleaning of a restaurant bar and its toilet facilities, a defective sewage distribution box leaked raw wastewater down the cliff, where the facilities were located. The wastewater emerged in the sand, where the people were contaminated by direct contact with bare skin, resulting in the skin-rash outbreak.

Beach sand may act as a reservoir for various microorganisms, such as enteric pathogens. The fecal pathogens potentially present in this environment tend to be considered a great risk to human health, especially for those with advanced age, diabetes, immunodepression (transient or permanent), and respiratory problems [4,7,8,12]. Contact with contaminated sand either by a dermal route, ingestion, or accidental inhalation [3,4,7,11] can cause intestinal infections, verminoses, and skin diseases, as well as the exacerbation of allergies, causing a profound impact on the population’s life quality [5,8,13]. Many of these pathogens originate from human or animal feces [14]. When introduced into the environment, they can persist for long periods; even if their concentration is low, these pathogens can still be considered harmful to human health because some of the pathogens have low infectious doses [15]. Sand is no exception, as described in Romão et al. [16], where the fecal contamination indicators in sand remained viable for approximately three months following an extreme weather event on the island of Madeira, Portugal.

In addition to possible contamination generated by contaminated seawater at times of rough seas or during high tides [12,17,18], beach sand can be contaminated by human or animal feces in several ways. Usually, contamination events arise from combined sewer overflows or discharges of wastewater with inadequate or non-existing treatment. Animal feces can be classified as being from domestic or feral animals, with the most common ones being from dogs, cattle and other ruminants, some wild animals, and birds (such as seagulls) [19,20,21,22,23,24,25,26].

Human feces and untreated wastewater are classified as highly dangerous for human exposure. This is because this type of feces has a greater potential for disease transmission to humans (greater compatibility and transmissibility of pathogens) compared to exposure to animal feces [27,28,29,30,31]. However, this ideology is based on the species barrier principle: each organism is more susceptible to its own set of disease-causing pathogens [32]. It should be noted that animal feces have a higher overall dominance, considering that these are usually less controlled than human ones, especially in rural areas [33,34].

The contamination type can be classified as point or diffuse sources. Point sources are easy to identify and manage, as the contamination reaches the watercourse in one concentrated place. Diffuse sources of contamination (also known as non-point sources) are usually more difficult to characterize and address, given their multiple origins [6,18,19,20,35,36,37,38].

Despite the difficulties, characterization of the contamination becomes crucial in management and remediation. Knowing the dominant sources of fecal contamination allows researchers and managing authorities to undertake remediation measures as well as identify the main risks to public health from these exposure routes [5,23].

Microbial source tracking (MST) is a molecular diagnostic approach for tracking fecal contamination sources at the animal-species level, even in cases of diffuse origin [8,17,23,31,32,36,37,39,40,41,42,43,44]. This is based on the hypothesis that there are host-associated microorganisms that each species has in their intestinal tract [17,19,20,23,32,35,36,45]. For example, *Bacteroidetes*, commensal bacteria prevalent in the intestinal microbiota of mammals, are mostly strict anaerobes adapted to living in the intestinal tract. These are one of the most selected bacteria for MST because they have a cell density greater than 10^10^/g of fecal matter and exhibit host specificity [32]. They can exceed *Escherichia coli* (*E. coli*) concentrations, especially when associated with the human large intestine [32,46]. Using specific marker gene(s) for these bacteria, it is possible to identify the sources of fecal contamination present in the sand and subsequently adopt and implement corrective measures to minimize the risks for public health and the environment [36,37].

### The Case under Study

A supratidal sand sampling campaign was conducted on 20 August 2020, on the Azorean beach Prainha (Terceira Island, Azores, Portugal) resulted in the detection of high levels of FIB, of unknown origin in 9 of the 10 sampling sites tested along the beach. The samples exceeding the local standards used in sand monitoring underwent MST analyses. This study was designed to identify and resolve the cause of the fecal indicator bacteria (FIBs) exceedance at Prainha Beach. Currently, the threshold values used in Portugal for sand are 10 CFU/g of enterococci and 25 CFU/g of *E. coli* based on Sabino et al. [47] and 100 coliform bacteria based on Brandão et al. [2]. As they were willing to understand the cause of the problem, the municipality engaged the local health protection services and national health authorities. Together, this institutional consortium assessed the following situation: several sources were identified as potential contamination sources: (a) run-off from mountains on the north coast, (b) avian contamination by seagulls, (c) direct beach contamination by users and dog walking, (d) urban grey-waters infiltration via broken draining installations or storm-related overflow, and (e) upwelling from the water table—sampling site 11. Several remediation measures were undertaken to allow beach users to use the beach once more, followed by the molecular analysis of the coastal sand samples to determine the likely sources of this contamination event.

## 2. Materials and Methods

Prainha beach is the only urban artificial sandy beach in Angra do Heroísmo (GPS: 38°39′13.9″ N 27°13′12.0″ W). It is in the Bay of Angra and is one of the main tourist sites, especially in the summer season. Visitors can swim on this beach located at the historic city center, which is a UNESCO world heritage site. The beach is bordered by the Bay of Angra, the marina, and the city wall. With the amount of hotel and restaurant services in the area, as well as the cultural offerings, it is a place of choice for residents and tourists, contributing to the economic growth of local residents. Tourist entertainment companies are located between Prainha and the marina in order to reach their target audience with greater accessibility. The whole area is frequented daily by approximately 300 to 400 users.

### 2.1. Collection and Preservation of the Sand Samples

**Figure 1:**
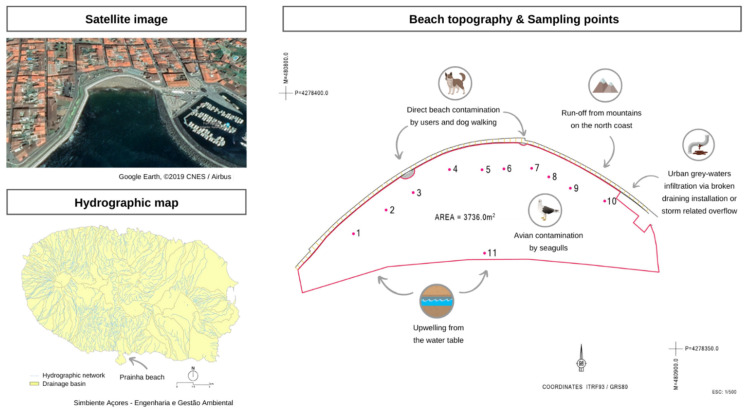
Prainha beach—satellite image from Google Earth, ©2019 CNES/Airbus [48] and topography with an indication of the sampling sites 1 to 11 (supplied by the municipality). The hydrographic map of the island is also included to show the possible runoffs that could have an impact on the beach [49].

In August 2019, there was an FIB exceedance in Prainha beach, with coliform ranging between 166 and >201 MPN/g, *E. coli* ranging between 28 and >201 MPN/g, and enterococci ranging between 10 and 201 MPN/g at different sampling sites of the beach. To identify the contamination dimension and origin, 11 sand samples were collected at several sites along the beach, according to the map displayed in Figure 1 showing 10 sites of supratidal sands and site 11 in the shoreline (close to an outfall of city stormwater overflow). The sand was analyzed for standard microbiological parameters (coliform bacteria, *E. coli*, and enterococci) and by an MST approach.

**Figure 2:**
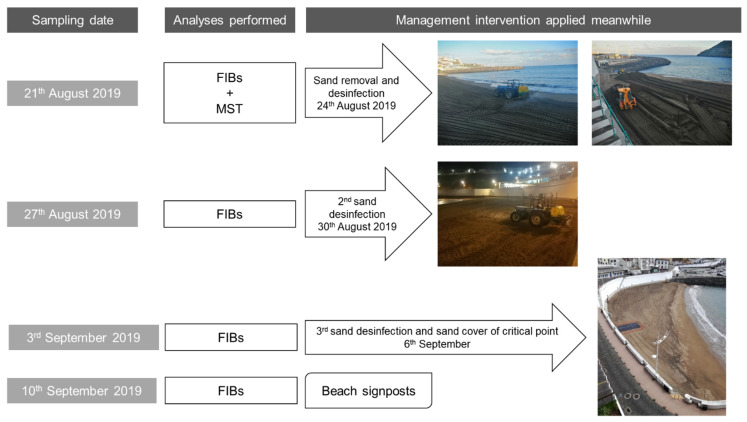
Sampling campaigns and interventions performed throughout the time.

Sampling took place in depths of up to 10 cm from the surface into a sterile container, according to Brandão et al. [2]. Samples were protected from sunlight and transported to the laboratory on ice. After homogenizing the sand, a part of each sample was analyzed immediately, and a portion was kept in a freezer at −20 °C to allow posterior analyses. After the first sampling campaign, several interventions started to be applied to restore sand quality to allow beach use. The various sampling campaigns and management interventions are summarized in Figure 2.

### 2.2. Microbiological Parameters

For detection of the FIBs, the methodology selected was previously implemented by Sabino et al. [47], where 50 g of sand from each sample was extracted in 500 mL of distilled water by shaking the mixture vertically with a rotation of 100 rpm for 30 min. The analyses followed, using Quanti-Tray^®^ systems from IDEXX^TM^ (IDEXX, Westbrook, MN, USA) to determine enterococci’s most probable number (MPN), Enterolert^®^ in 10 mL of the eluent, according to the manufacturer’s instructions for water samples. The same procedure was applied for total coliforms and *E. coli* using the MPN Colilert^®^ (also from IDEXX^TM^).

### 2.3. DNA Extraction from Sand Samples

Until the beginning of this work, there was no established standard method for extracting genomic DNA from the microbes present in sand samples for microbial source tracking analyses, using commercial DNA extraction kits. The approaches decided by the team consisted of (*Approach A*) washing the sediments followed by successive filtration steps combined with PCR detection 1 in Section 2.4. Subsequently, while performing the geographical validation of more MST, another approach was used based on the direct extraction of the DNA from a frozen sand sample—*Approach B* combined with PCR detection 2 described in Section 2.4.

*Approach A:* A portion of each sand sample was used to extract the DNA by washing 20 g of sand with 50 mL of water, followed by 30 min of orbital agitation. The extraction water was then filtered through 0.45 μm pore size polycarbonate membranes (diameter 47 mm) (Whatman^®^, Maidstone, UK). The DNA was subsequently extracted from the filter membranes using an Aquadien (BioRad, Hercules, CA, USA) kit, using the manufacturer’s protocol.

*Approach B:* This approach was used to extract the DNA from the frozen sand samples. A sample of 3 g of each sand sample was extracted using DNeasy Power Water Kit (Qiagen, Hilden, Germany) with some adaptations to increase the DNA yield (direct use of sand instead of the membrane, and 10 min vortex of the sample instead of the usual 5 min vortexing). Notes: (1) The DNeasy Power Water Kit (Qiagen) was first used according to the manufacturer’s instructions and then subjected to slight modifications, namely the amount of sand and the vortexing time. (2) This approach was also subsequently applied to fresh sand samples with success, for which, 1.5 g of sand was enough (data not shown).

### 2.4. Microbial Source Tracking (MST) Analyses

Species-specific *Bacteroides* primers used in this study were selected from the literature and consulting with experts (Table 1). All the PCR conditions were tested and optimized, and all the primers were validated using local fecal samples of all five biological sources tested. This quality assurance was done on the premise that a geographical validation of MST primers is recommended before use [40,50]. According to the local authorities, beach sand contamination from run-off from mountains on the north coast could be identified by the presence of fecal matter from cows, other ruminants, and eventually pigs. The other species selected were based on the common uses of the beach—human activities and dog walking, and seagulls which are typically coastal or inland species.

#### 2.4.1. Primers

Each primer set (Table 1) was validated by conventional PCR for host sensitivity and specificity. For that purpose, fresh fecal samples were collected from various individuals from different locations of Portugal. This was performed because the geographical location and individual characteristics might affect the composition of the gut flora and, in turn, the presence of enteric bacteria associated with MST markers. The fecal samples used in this study belonged to humans, domestic animals (dogs and cats), livestock (donkeys, horses, cows, goats, sheep, and pigs), and birds (canaries, seagulls, chickens, ducks, and turkeys). In order to ensure some variety in the biological sources tested to robustly validate primers within the country, a total of thirty-six fecal samples were used: three belonged to humans; eight to domestic animals (four from dogs and four from cats); eighteen from livestock (one from donkeys, five from horses, five from cows, three from goats, two from sheep, and two from pigs); and seven from birds (one from canaries, two from seagulls, two from chickens, one from a duck, and one from a turkey).

#### 2.4.2. DNA Extraction for MST Validation

DNA extraction from fecal samples was performed using the DNeasy PowerWater Kit (14900-100-NF) from Qiagen. The protocol associated with the Qiagen kit was adapted: instead of working with filters, a small amount of the fecal sample of interest was placed directly into the PowerWater DNA Bead Tube, together with 1 mL of the PW1 solution. After extraction, a NanoDrop One (Thermo Fisher Scientific) was used to verify and evaluate the quantity and quality of DNA obtained.

The volume of DNA to be used in the PCR reactions was determined based on its concentration. Usually, 2 µL of DNA was enough to obtain 20 to 30 ng of DNA per PCR reaction. However, in the case of DNA with a lower concentration, 3 µL was used as template to compensate.

#### 2.4.3. PCR Detection 1—Fresh Coastal Sand Samples

At the time of this contamination event, we had available and validated human and cow molecular markers from the previous work of Teixeira et al. [54]. Following the DNA extraction (using *Approach A*) from fresh coastal sands, samples were tested by qPCR for human and cow contamination using the conditions previously described in Teixeira et al. [54]. Briefly, for the HF183/Bac287 Real-Time qPCR assay, 10 μL reaction mixtures containing 1× SsoAdvanced Universal Probe Supermix (Biorad, 0.2 mg/mL BSA (bovine serum albumin), 0.5 μM of each primer, 80 nM (FAM)-labelled probe, and 2 μL of DNA template or molecular-grade water (no-template control—NTC) were prepared. The thermal cycling conditions were 10 min at 95 °C followed by 40 cycles of 15 s at 95 °C and 60 s at 60 °C [51]. For the CowM2 qPCR assay, 10 μL reaction mixtures containing 1× SsoAdvanced Univ Probes Supermix (Biorad), 0.2 mg/mL BSA, 1 μM of each primer, 80 nM (FAM)-labelled probe, and 2 μL of DNA template or molecular-grade water (NTC) were prepared. The thermal cycling conditions were 10 min at 95 °C followed by 40 cycles of 15 s at 95 °C and 60 s at 60 °C [53].

#### 2.4.4. PCR Detection 2—Fecal Samples for MST Validation and Frozen Coastal Sand Samples

Meanwhile, more molecular markers were geographically validated (Table 1), and a new analysis was performed in the previously collected samples that had been kept at −20 °C. DNA obtained from the frozen sand samples (using *Approach* *B*) was analyzed with the primers indicated in Table 1 by conventional PCR. PCR reactions of 25 μL PCR were composed of 1× PCR buffer (BIOTAQ DNA polymerase), 1 U of Taq polymerase (BIOTAQ DNA polymerase), 3 mM of MgCl_2_, 1 mM of dNTPs, 1 μM of each primer pair, and 20–30 µg of DNA template.

PCR negative controls were included in each set of PCR reactions to monitor contamination. Simultaneously, positive controls (DNA extracted from fecal samples) were also included in each PCR plate.

The amplification occurred in a TPersonal thermal cycler (Biometra, Analytik Jena AG, Jena, Germany). PCR cycling conditions were as follows: initial denaturation at 95 °C for 5 min; 40 cycles of (1) denaturation step at 94 °C for 45 s, (2) annealing step for each primer pair, at temperatures indicated in Table 1 for 45 s, and (3) elongation step at 72 °C for 1 min; and a final extension at 72 °C for 5 min. PCR products were visualized using 1% agarose gels, stained with GelRed^®^ Nucleic Acid Stain (Biotium), and using a 100 bp DNA ladder (PanReac AppliChem, ITW Reagents). The visualization of the gel was performed under a UV-light transilluminator (UVITEC Cambridge, Cambridge, UK).

The full detailed protocol for MST analysis of sand as designed for the current study can be found in Supplementary Materials.

## 3. Results

### 3.1. Management and Remediation Measures

The supratidal sand sampling campaign that took place on 21 August 2020 at beach Prainha resulted in the detection of high levels of FIB in 9 of the 10 sampling sites tested along the beach (Table 2, column “FIB results” highlighted with bold text). In order to protect the health of the beach users, three days before the following sampling campaign (24 August), a drastic intervention took place with the removal of 400 m^3^ of sand and spraying of the entire beach (3736 m^2^) with 1000 L of chlorine (6.5% sodium hypochlorite aqueous solution). The second sampling campaign that took place on 27 August, where a wet sand sample was also analyzed (sampling site 11), did not show any high levels of FIB. Only a low count (4 MPN/g) for coliforms in sampling site 2 suggested a possible new contamination after the beach sand treatment.

One week later (3 September sampling campaign), sampling sites 1 to 10 were tested once again following a spraying event (30 August) with 600 L of a 6.5% sodium hypochlorite solution along the entire length of the beach. The FIB results were below the recommendations used locally (10 MPN/g, 25 MPN/g, and 100 MPN/g, of enterococci, *E. coli*, and coliform bacteria, respectively) for the sampling sites 1 and 3 to 10; however, site 2 exceeded the recommendation for coliforms. This last result supported the previous suspicion that a de novo contamination was taking place after the beach spraying.

Unable to resolve the recurrent sand contamination immediately, and to distinguish fecal deposits from contamination upwelling, the solution was to physically eliminate the possible contamination sources by covering a 4 m × 4 m area (at sampling site 2) of the beach with an impermeable plastic film, as shown in Figure 2 (dark grey patch on the sand). Moreover, access to the beach was restricted to differentiate upwelling from deposition. A new spraying event took place on the entire beach length with 400 L of chlorine (6.5% sodium hypochlorite aqueous solution) on 6 September. The 10 September sampling campaign (at sampling sites 2, 3, 4, 9, and 10) excluded a possible upwelling origin of the contamination. The local authorities assumed that the contamination could come from dog walking after regular beach use hours, and a decision to discourage that activity for the local inhabitants was made and signage prohibiting dog walking was put in place.

### 3.2. MST Markers Validation

Until the beginning of this work, there was no standard or most suitable method established to extract genomic DNA of the microbes present in sand samples, as is conducted for water MST analysis. Therefore, at the time of the contamination episode, the approach followed consisted of washing the sand samples followed by filtration and subsequent use of a DNA extraction kit (*Approach A*). However, we verified that a higher yield and quality of the extracted DNA was obtained with *Approach B*, which granted more confidence in the results obtained by PCR. This approach was later applied to new fresh sand samples from this beach and others with success, for which we verified that 1.5 g of sand was enough to obtain a good yield.

Geographic validation of the selected MST markers was performed using DNA from local fecal samples to avoid errors in the interpretation of results associated with an MST study, such as cases of cross-reactivity. The quality control results are summarized in Table 3. We observed that all primers tested were successfully validated. There was only one case of cross-reactivity detected, which referred to the DNA sample extracted from a fresh fecal sample from a horse, which was amplified with the ruminant primer set.

### 3.3. Microbiological and MST Results

As already mentioned above, the microbiological parameters for the threshold limits currently used in Portugal for sand are 10 CFU/g of enterococci and 25 CFU/g of *E. coli* based on Sabino et al. [47] and 100 coliform bacteria based on Brandão et al. [2].

Analyzing Table 2, it is noticeable that in the case of samples collected on August 21, all the samples, except for sampling site 1, showed at least one FIB parameter higher than what is recommended (highlighted in bold in Table 2). The following sampling campaign on September 3 also indicated fecal contamination in one of the sampling sites (highlighted in bold in Table 2), and some other sampling sites showed the presence of FIBs (sites 3, 4, and 10). The final campaign that took place on 10 September 2019 did not indicate any FIB contamination.

**Figure 3:**
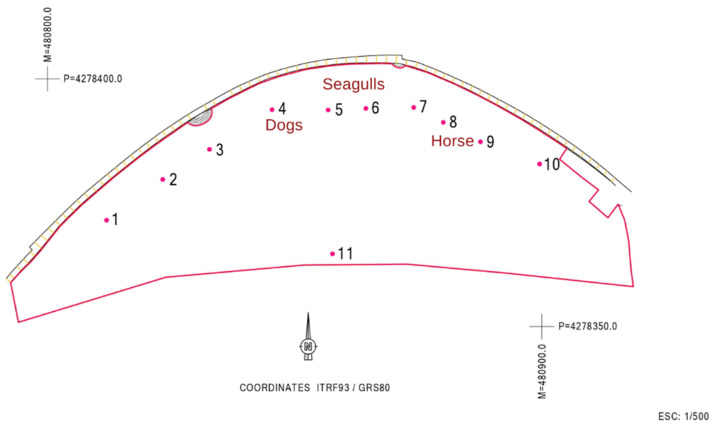
Assignment of the respective contamination sources to each of the sampled sites.

Regarding the MST approach, the dog marker gene was detected only on sampling location 4; the seagull 2 marker gene was detected in sampling location 5, and a positive signal for the ruminant marker gene was detected in sampling locations 8 and 9. No consistency was thus found in terms of contamination sources for the sites tested. Figure 3 shows the location of the sites that were positive for the different MST markers.

## 4. Discussion

Bacteriological analysis in sand has a similar objective to FIB enumeration in water, as it aims to detect and quantify bacteria (total coliforms, *E. coli*, and enterococci). Other bacterial and non-bacterial parameters may be used to indicate other forms of contamination, such as skin and hair shedding, and endemic species of interest in public health protection [8]. Fecal contamination, however, is the best-studied aspect in sand quality because it may not only represent a direct exposure route but also a diffuse source of contamination in water quality [10,55].

It is important to carefully monitor the quality of the sand being tested and not just the water to avoid outbreaks like the one described in Brandão et al. [11]. In that episode, a deteriorated sewage distribution box was the cause of an outbreak in 30 people, in which sand was the proven fomite. Events like this were the basis of a study by Heaney et al. [56], where an epidemiological study compared the health effects of handling sand in the biased scenario of a beach with a nearby, publicly owned, treatment-works outfall. In that study, handling the beach sand contaminated with *Enterococcus* led to an increase by over two-fold of gastrointestinal illness cases, with the highest incidence being in those who buried themselves in sand, with 3.3 times more cases of gastrointestinal illness than those of the reference group. It seems we need to be more assertive in assessing the quality of a beach that is used by all kinds of people, including toddlers, who play with sand, including the elderly and immunologically compromised individuals (transiently or permanently), as well as those with cystic fibrosis or diabetes.

MST approaches have been used to distinguish between the sources of fecal pollution in stormwater, recreational water, and surface waters worldwide, with high degrees of specificity and sensitivity [19,22,23,24,25]. In the study of Nevers et al. [18], intertidal sand, sediment, and overlying water at three shoreline sites and two associated rivers along an extended freshwater shoreline were analyzed. The parameters evaluated were FIB, two MST markers (Gull2 and HF183), and the targeted metagenomic 16S rRNA gene. They were able to establish a relationship between bacteria in the sand, sediment, and overlying water; they concluded that FIB and MST markers were effective estimates of short-term conditions at these locations, while bacterial communities in sand and sediment reflected longer-term conditions.

It may also be time to examine climate change impacts on fecal contamination of unknown origin since extreme storm events because of climate change have reportedly resulted in destructive land and mudslides and runoff that contaminate the beach sand [16].

After reviewing all the information that is summarized in Table 2 and from the remediation and management measures, it was possible to assign the respective contamination source in the different locations (Figure 3). The dogs’ markers at site 4 support the authorities’ suspicions and reinforce the need to avoid this kind of practice that contributes to beach contamination. The seagulls’ marker detection was not a surprise, given that they are natural habitants of these environments. Sampling site 11 is close to an outfall of city storm waters overflow, which was one of the possible contamination sources upwelling from the water table, and was therefore locally tested for FIB, but returned no useful information.

As mentioned in the results, the primers used for the ruminants’ marker gene gave a positive result in sampling locations 8 and 9. However, the cow marker gene (one of the possible ruminants) was not detected. It is possible that the primers used in this study for the ruminant marker gene may have cross-reactivity with horses or other animals [19,40,57,58,59]. Keeping this in mind, the team looked for the possible cultural events that had taken place near the beach at that time and discovered that throughout the summer, a horse-drawn carriage for sightseeing had been parked for some time near sites 8 and 9.

*Enterococcus*, *E. coli*, and *Bacteroides* are three types of bacteria, which belong to different phyla [60], which are usually found in the intestines of warm-blooded animals [61]. Therefore, after a recent episode of fecal contamination, one can expect to find them all. However, the results obtained here highlight that despite the microbiological parameters being high in some sampling points, the DNA from *Bacteroides* was not detected, thus not allowing the assignment of the fecal contamination source. These results must be examined carefully, since negative results in the PCRs do not mean complete absence of bacteria, as can be verified in the microbiological results for FIB growth, also presented in Table 2. This just means that the primers used did not result in amplification, indicating the absence of the target DNA. Several other factors must also be kept in mind: (1) we may not have used the most adequate primers to identify the DNA region of the *Bacteroides* species present; (2) there might be other contamination sources besides the ones examined, e.g., from rodents; (3) the survival time of *Bacteroides* outside the intestine is much shorter (ca. 48 h) when compared to *Enterococcus* (several weeks) or *E. coli* (6 h) [62]. In this study, it is more probable that the last reason (the survival time) is behind the results obtained since the MST analysis started sometime after the microbiological ones. This led us to be aware that the MST analyses should start preferably within the 24 h after sampling.

The several steps that were taken to decontaminate the sand have been shown to be efficient. The detection of the horse- and dog-associated marker gene highlights the need to protect beaches from animal walking, which contributes to the high levels of FIBs subsequently detected. Moreover, further implementation of dog-walking restrictions and advisory signs helped to mitigate part of the contamination problem.

## 5. Conclusions

The new WHO guidelines for recreational water quality were released last July [8], recommending sand analysis for enterococci and fungi, with, respectively, provisional limits of 60 CFU/g and a guiding value of 89 CFU/g. Although researchers sometimes use the traditional fecal indicators *E. coli* and *Enterococcus* to monitor sand quality [63,64,65], this methodology does not allow the biological source of the contamination to be inferred. The next natural step is for these recommendations to slowly be integrated into regulations. In this case, we show that MST provides a valuable tool to investigate potential fecal pollution sources of both sand and water.

We examined the origin of the sand contamination of a coastal beach in the Azores. There are not many episodes of contaminated sand reported but this study shows that the microbial source tracking approach is a useful method in determining the biological source of the fecal contamination, demonstrating that, in this case, it was attributed to multiple sources. This is a very important approach to manage pollution episodes, as it sheds light upon the possible biological contributions that can often be readily contained by local public health officers.

Another important and practical aspect of this study is the course of disinfection that took place at the beach to lower the levels of fecal indicator microbes in the sand.

Recognizing that beaches suffer contamination events, this study shows the importance of holistic management of the beaches worldwide that should go beyond the already established water quality monitoring for FIB, putting forth evidence of the need to also monitor sands to avoid public health problems.

## Figures and Tables

**Table 1:** List of microbial source tracking primers used and their respective sequence, annealing temperatures, and conditions tested, as well as the original reference that described them.

Target	Primers’ Names	Sequence (5′–3′)	Conventional PCR Annealing Temperature	Tested by Probe-Based qPCR	References
Humans	HF183F BacR287	F: ATCATGAGTTCACATGTCCG R: CTTCCTCTCAGAACCCCTATCC P: FAM–CTAATGGAACGCATCCC–MGBEQ	53 °C	YES	[51]
Dogs	DF113F DF472R	F: ATCTCAAGAGCACATGCAA R: AATAAATCCGGATAACGCTC	53 °C	NO	[21]
Seagulls	Gull-2F Gull-2R	F: TGCATCGACCTAAAGTTTTGAG R: GTCAAAGAGCGAGCAGTTACTA	53 °C	NO	[52]
Ruminants	RUM_CF128F RUM_Bac708R	F: CCAACYTTCCCGWTACTC R: CAATCGGAGTTCTTCGTG	60 °C	NO	[46]
Cows	CowM2F CowM2R	F: CGGCCAAATACTCCTGATCGT R: GCTTGTTGCGTTCCTTGAGATAAT P: FAM–AGGCACCTATGTCCTTTACCT CATCAACTACAGACA–MGBEQ	ND	YES	[53]
Cows	CowM3F CowM3R	F: CCTCTAATGGAAAATGGATGGTATCT R: CCATACTTCGCCTGCTAATACCTT	53 °C	NO	[53]
Pigs	Bac41F Bac163R	F: GCATGAATTTAGCTTGCTAAATTTGAT R: ACCTCATACGGTATTAATCCGC	60 °C	NO	[22]

Legend: ND: not determined for this approach. Note: The bovine primers were used only when samples: (1) were positive for ruminants (using conventional PCR) or (2) were those analyzed by qPCR.

**Table 2:** Results of the microbiological tests and microbial source tracking (MST) of the sand samples collected on several dates throughout this study.

Sampling Date	21 August 2019						27 August 2019	3 September 2019	10 September 2019
Sampling Sites	Fecal Indicator Bacteria Results	MST Results					Fecal Indicator Bacteria Results		
		Humans	Dogs	Seagulls	Ruminants	Bovine			
Site 1	Coliform bacteria, 1 MPN/g *Escherichia coli*, 1 MPN/g *Enterococcus* spp., <1 MPN/g	NEG	NEG	NEG	NEG	ND		Coliform bacteria, <1 MPN/g *Escherichia coli*, <1 MPN/g *Enterococcus* spp., <1 MPN/g	
Site 2	**Coliform bacteria, 201 MPN/g** ***Escherichia coli*** **, 84 MPN/g** ***Enterococcus* spp., 201 MPN/g**	NEG	NEG	NEG	NEG	NEG	Coliform bacteria, 4 MPN/g *Escherichia coli*, <1 MPN/g *Enterococcus* spp., <1 MPN/g	**Coliform bacteria, 102 MPN/g** *Escherichia coli*, <1 MPN/g *Enterococcus* spp., <1 MPN/g	Coliform bacteria, <1 MPN/g *Escherichia coli*, <1 MPN/g *Enterococcus* spp., <1 MPN/g
Site 3	**Coliform bacteria, >201 MPN/g** ***Escherichia coli*** **, >201 MPN/g** ***Enterococcus* spp., 201 MPN/g**	NEG	NEG	NEG	NEG	ND		Coliform bacteria, 4 MPN/g *Escherichia coli*, <1 MPN/g *Enterococcus* spp., <1 MPN/g	Coliform bacteria, <1 MPN/g *Escherichia coli*, <1 MPN/g *Enterococcus* spp., <1 MPN/g
Site 4	Coliform bacteria, 14 MPN/g *Escherichia coli*, 1 MPN/g ***Enterococcus* spp., 10 MPN/g**	NEG	**POS**	NEG	NEG	NEG		Coliform bacteria, 9 MPN/g *Escherichia coli*, <1 MPN/g *Enterococcus* spp., 9 MPN/g	Coliform bacteria, <1 MPN/g *Escherichia coli*, <1 MPN/g *Enterococcus* spp., <1 MPN/g
Site 5	**Coliform bacteria, >201 MPN/g** ***Escherichia coli*** **, 110 MPN/g** ***Enterococcus* spp., 74 MPN/g**	NEG	NEG	**POS**	NEG	ND	Coliform bacteria, <1 MPN/g *Escherichia coli*, <1 MPN/g *Enterococcus* spp., <1 MPN/g	Coliform bacteria, <1 MPN/g *Escherichia coli*, <1 MPN/g *Enterococcus* spp., <1 MPN/g	
Site 6	**Coliform bacteria, 166 MPN/g** ***Escherichia coli*** **, 63 MPN/g** ***Enterococcus* spp., 51 MPN/g**	NEG	NEG	NEG	NEG	ND		Coliform bacteria, <1 MPN/g *Escherichia coli*, <1 MPN/g *Enterococcus* spp., <1 MPN/g	
Site 7	Coliform bacteria, 5 MPN/g *Escherichia coli*, 1 MPN/g ***Enterococcus* spp., 130 MPN/g**	NEG	NEG	NEG	NEG	NEG		Coliform bacteria, <1 MPN/g *Escherichia coli*, <1 MPN/g *Enterococcus* spp., <1 MPN/g	
Site 8	**Coliform bacteria, >201 MPN/g** *Escherichia coli*, 12 MPN/g ***Enterococcus* spp., 24 MPN/g**	NEG	NEG	NEG	**POS**	NEG		Coliform bacteria, 1 MPN/g *Escherichia coli*, <1 MPN/g *Enterococcus* spp., 1 MPN/g	
Site 9	**Coliform bacteria, >201 MPN/g** ***Escherichia coli*** **, 28 MPN/g** ***Enterococcus* spp., 28 MPN/g**	NEG	NEG	NEG	**POS**	NEG	Coliform bacteria, <1 MPN/g *Escherichia coli*, <1 MPN/g *Enterococcus* spp., <1 MPN/g	Coliform bacteria, <1 MPN/g *Escherichia coli*, <1 MPN/g *Enterococcus* spp., <1 MPN/g	Coliform bacteria, <1 MPN/g *Escherichia coli*, <1 MPN/g *Enterococcus* spp., <1 MPN/g
Site 10	**Coliform bacteria, 201 MPN/g** *Escherichia coli*, 13 MPN/g *Enterococcus* spp., 4 MPN/g	NEG	NEG	NEG	NEG	ND		Coliform bacteria, 2 MPN/g *Escherichia coli*, 1 MPN/g *Enterococcus* spp., <1 MPN/g	Coliform bacteria, <1 MPN/g *Escherichia coli*, <1 MPN/g *Enterococcus* spp., <1 MPN/g
Site 11							Coliform bacteria, <1 MPN/g *Escherichia coli*, <1 MPN/g *Enterococcus* spp., <1 MPN/g		

Legend: NEG—negative result (absence of DNA from the tested source); POS—positive result (detection of the DNA from the tested source). ND—not determined. Highlighted with bold text: high levels of FIB.

**Table 3:** Summarized table of the amplifications associated with each molecular marker selected. Represented with “✓” are the samples that, when amplified by the marker, presented a band in the region of interest, and represented with “✗” are those that did not present bands in that region. ND—not determined for the primers. The symbol “⚠” indicates that there was evidence of cross-reactivity when the marker was tested for a given fecal sample.

Target	Primers’ Names	Humans	Domestic Animals		Livestock						Birds				
			Cat	Dog	Donkey	Horse	Cow	Goat	Sheep	Pig	Canaries	Seagull	Chicken	Duck	Turkey
Humans	HF183F BacR287	**✓**	ND	**✗**	**✗**	**✗**	**✗**	**✗**	**✗**	**✗**	ND	**✗**	**✗**	**✗**	**✗**
Dogs	DF113F DF472R	**✗**	**✗**	**✓**	ND	**✗**	**✗**	**✗**	**✗**	**✗**	**✗**	ND	**✗**	ND	ND
Seagulls	Gull-2F Gull-2R	**✗**	ND	**✗**	ND	**✗**	**✗**	**✗**	**✗**	**✗**	**✗**	**✓**	**✗**	**✗**	**✗**
Ruminants	RUM_CF128F RUM_Bac708R	**✗**	ND	**✗**	**✗**	⚠	**✓**	**✓**	**✓**	**✗**	ND	ND	**✗**	ND	ND
Cows	CowM3F CowM3R	**✗**	ND	**✗**	**✗**	**✗**	**✓**	**✗**	**✗**	**✗**	ND	ND	ND	**✗**	ND
Pigs	Bac41F Bac163R	**✗**	ND	**✗**	**✗**	**✗**	**✗**	**✗**	**✗**	**✓**	ND	ND	**✗**	ND	ND

## Data Availability

Not applicable.

## Supplementary Materials

The following supporting information can be downloaded at: https://www.mdpi.com/article/10.3390/ijerph19137934/s1, Protocol for MST analysis of sand as designed for the current study.

## References

[1] Pinto A.B., Oliveira A.J.F.C. (2011). Diversidade de microrganismos indicadores utilizados na avaliação da contaminação fecal de areias de praias recreacionais marinhas: Estado atual do conhecimento e perspectivas. Mundo Saúde.

[2] Brandão J., Wergikosky B., Rosado C., Noronha G., Veríssimo C., Falcão L., Giraldes A., Simões M., Rebelo H. (2002). Qualidade Microbiológica de Areias de Praias Litorais—Relatório Final.

[3] Brandão J., Silva C., Alves C., Cunha M., Moura I., Veríssimo C., Wergikoski B., Parada H., Falcão L., Barroso M. (2008). Monitorização da qualidade das areias em zonas balneares—Época balnear de 2008.

[4] Brito S., Sabino R., Veríssimo C., Silva S., Valério E., Brandão J. (2019). Fungos em areias e águas costeiras e interiores em Portugal—Relevância para a saúde humana e bem-estar. Obs. Bol. Epidemiol..

[5] Lescreck M.C., Petroni R.G.G., Cortez F.S., Santos A.R., Coutinho P.O., Pusceddu F.H. (2016). Análise da qualidade sanitária da areia das praias de Santos, litoral do estado de São Paulo. Eng. Sanit. E Ambient..

[6] Safaie A., Weiskerger C.J., Nevers M.B., Byappanahalli M.N., Phanikumar M.S. (2021). Evaluating the impacts of foreshore sand and birds on microbiological contamination at a freshwater beach. Water Res..

[7] Weiskerger C.J., Brandão J. (2020). Fungal contaminants in water and sand: A new frontier for quantitative microbial risk assessment. Curr. Opin. Environ. Sci. Health.

[8] World Health Organization (2021). Guidelines on Recreational Water Quality: Volume 1 Coastal and Fresh Waters.

[9] World Health Organization (2003). Guidelines for Safe Recreational Water Environments: Volume 1 Coastal and Fresh Waters.

[10] Solo-Gabriele H.M., Harwood V.J., Kay D., Fujioka R.S., Sadowsky M.J., Whitman R.L., Wither A., Caniça M., da Fonseca R.C., Duarte A. (2015). Beach sand and the potential for infectious disease transmission: Observations and recommendations. J. Mar. Biol. Assoc. UK.

[11] Brandão J., Albergaria I., Albuquerque J., José S., Grossinho J., Ferreira F., Raposo A., Rodrigues R., Silva C., Jordao L. (2020). Untreated sewage contamination of beach sand from a leaking underground sewage system. Sci. Total Environ..

[12] Gast R.J., Gorrell L., Raubenheimer B., Elgar S. (2011). Impact of erosion and accretion on the distribution of enterococci in beach sands. Cont. Shelf Res..

[13] Brandão J., Gangneux J., Arikan-Akdagli S., Barac A., Bostanaru A., Brito S., Bull M., Çerikçioğlu N., Chapman B., Efstratiou M. (2021). Mycosands: Fungal diversity and abundance in beach sand and recreational waters—Relevance to human health. Sci. Total Environ..

[14] Ko H.Y., Cho K., Park S., Kim J.H., Kang J.-H., Jeong Y.S., Choi J.D., Sin Y., Lee C., Ko G. (2018). Host-Specific Bacteroides Markers-Based Microbial Source Tracking in Aquaculture Areas. Microbes Environ..

[15] Pathogen Safety Data Sheets. https://www.canada.ca/en/public-health/services/laboratory-biosafety-biosecurity/pathogen-safety-data-sheets-risk-assessment.html.

[16] Romão D., Abreu R., Calado G., Freitas F., Rodrigues P., Ferreira C., Campos A., Temtem R., Freitas M., Andrade C. Madeira 2010—Aftermath of flashfloods and mudslides on bathing water quality indicators and on sand microbial levels. Proceedings of thePan-European Symposium on Water and Sanitation Safety Planning and Extreme Weather Events.

[17] Henry R., Schang C., Coutts S., Kolotelo P., Prosser T., Crosbie N., Grant T., Cottam D., O’Brien P., Deletic A. (2016). Into the deep: Evaluation of SourceTracker for assessment of faecal contamination of coastal waters. Water Res..

[18] Nevers M.B., Byappanahalli M.N., Nakatsu C.H., Kinzelman J.L., Phanikumar M.S., Shively D.A., Spoljaric A. (2020). Interaction of bacterial communities and indicators of water quality in shoreline sand, sediment, and water of Lake Michigan. Water Res..

[19] Ballesté E., Demeter K., Masterson B., Timoneda N., Sala-Comorera L., Meijer W.G. (2020). Implementation and integration of microbial source tracking in a river watershed monitoring plan. Sci. Total Environ..

[20] Gawler A.H., Beecher J.E., Brandão J., Carroll N.M., Falcão L., Gourmelon M., Masterson B., Nunes B., Porter J., Rincé A. (2007). Validation of host-specific Bacteriodales 16S rRNA genes as markers to determine the origin of faecal pollution in Atlantic Rim countries of the European Union. Water Res..

[21] Hussein K., Waines P., Nisr R., Glegg G., Bradley G. (2014). Development and use of Bacteroides 16S rRNA polymerase chain reaction assay for source tracking dog faecal pollution in bathing waters. Hydrol. Curr. Res..

[22] Mieszkin S., Furet J.-P., Corthier G., Gourmelon M. (2009). Estimation of Pig Fecal Contamination in a River Catchment by Real-Time PCR Using Two Pig-Specific *Bacteroidales* 16S rRNA Genetic Markers. Appl. Environ. Microbiol..

[23] Nguyen K., Senay C., Young S., Nayak B., Lobos A., Conrad J., Harwood V. (2018). Determination of wild animal sources of fecal indicator bacteria by microbial source tracking (MST) influences regulatory decisions. Water Res..

[24] Ryu H., Griffith J.F., Khan I.U.H., Hill S., Edge T.A., Toledo-Hernandez C., Gonzalez-Nieves J., Domingo J.S. (2012). Comparison of Gull Feces-Specific Assays Targeting the 16S rRNA Genes of *Catellicoccus marimammalium* and *Streptococcus* spp.. Appl. Environ. Microbiol..

[25] Shanks O.C., White K., Kelty C.A., Hayes S., Sivaganesan M., Jenkins M., Varma M., Haugland R.A. (2010). Performance Assessment PCR-Based Assays Targeting Bacteroidales Genetic Markers of Bovine Fecal Pollution. Appl. Environ. Microbiol..

[26] Xu Y., Li Z., Liu R., Liang H., Yu Z., Zhang H. (2020). Validation of Bacteroidales-based microbial source tracking markers for pig fecal pollution and their application in two rivers of North China. Front. Environ. Sci. Eng..

[27] Schoen M.E., Ashbolt N.J. (2010). Assessing Pathogen Risk to Swimmers at Non-Sewage Impacted Recreational Beaches. Environ. Sci. Technol..

[28] Schoen M.E., Soller J.A., Ashbolt N.J. (2011). Evaluating the importance of faecal sources in human-impacted waters. Water Res..

[29] Soller J.A., Bartrand T., Ashbolt N.J., Ravenscroft J., Wade T.J. (2010). Estimating the primary etiologic agents in recreational freshwaters impacted by human sources of faecal contamination. Water Res..

[30] Soller J.A., Schoen M.E., Bartrand T., Ravenscroft J., Ashbolt N.J. (2010). Estimated human health risks from exposure to recreational waters impacted by human and non-human sources of faecal contamination. Water Res..

[31] Zhang Q., Gallard J., Wu B., Harwood V.J., Sadowsky M.J., Hamilton K.A., Ahmed W. (2019). Synergy between quantitative microbial source tracking (qMST) and quantitative microbial risk assessment (QMRA): A review and prospectus. Environ. Int..

[32] Fujioka R.S., Solo-Gabriele H.M., Byappanahalli M.N., Kirs M. (2015). U.S. Recreational Water Quality Criteria: A Vision for the Future. Int. J. Environ. Res. Public Health.

[33] Delahoy M.J., Wodnik B., McAliley L., Penakalapati G., Swarthout J., Freeman M.C., Levy K. (2018). Pathogens transmitted in animal feces in low- and middle-income countries. Int. J. Hyg. Environ. Health.

[34] Penakalapati G., Swarthout J., Delahoy M.J., McAliley L., Wodnik B., Levy K., Freeman M.C. (2017). Exposure to Animal Feces and Human Health: A Systematic Review and Proposed Research Priorities. Environ. Sci. Technol..

[35] García-Aljaro C., Blanch A.R., Campos C., Jofre J., Lucena F. (2018). Pathogens, faecal indicators and human-specific microbial source-tracking markers in sewage. J. Appl. Microbiol..

[36] Griffith J., Layton B., Boehm A., Holden P., Jay J., Hagedorn C., McGee C., Weisberg S. (2013). The California Microbial Source Identification Manual: A Tiered Approach to Identifying Fecal Pollution Sources to Beaches.

[37] Hughes B., Beale D.J., Dennis P.G., Cook S., Ahmed W. (2017). Cross-Comparison of Human Wastewater-Associated Molecular Markers in Relation to Fecal Indicator Bacteria and Enteric Viruses in Recreational Beach Waters. Appl. Environ. Microbiol..

[38] Unno T., Staley C., Brown C.M., Han D., Sadowsky M.J., Hur H.-G. (2018). Fecal pollution: New trends and challenges in microbial source tracking using next-generation sequencing. Environ. Microbiol..

[39] Field K.G., Samadpour M. (2007). Fecal source tracking, the indicator paradigm, and managing water quality. Water Res..

[40] Harwood V.J., Staley C., Badgley B.D., Borges K., Korajkic A. (2014). Microbial source tracking markers for detection of fecal contamination in environmental waters: Relationships between pathogens and human health outcomes. FEMS Microbiol. Rev..

[41] Staley Z.R., Boyd R.J., Shum P., Edge T.A. (2018). Microbial Source Tracking Using Quantitative and Digital PCR To Identify Sources of Fecal Contamination in Stormwater, River Water, and Beach Water in a Great Lakes Area of Concern. Appl. Environ. Microbiol..

[42] Stoeckel D. (2005). Selection and application of microbial source tracking tools for water-quality investigations. Book 2: Collection of Environmental Data, Section A. Biological Science—Techniques and Methods.

[43] U.S. Environmental Protection Agency, Office of Water, Office of Science and Technology, Health and Ecological Criteria Division (2014). Overview of Technical Support Materials: A Guide to the Site-Specific Alternative Recreational Criteria TSM Documents.

[44] Vadde K.K., McCarthy A.J., Rong R., Sekar R. (2019). Quantification of Microbial Source Tracking and Pathogenic Bacterial Markers in Water and Sediments of Tiaoxi River (Taihu Watershed). Front. Microbiol..

[45] Ahmed W., Gyawali P., Feng S., McLellan S.L. (2019). Host Specificity and Sensitivity of Established and Novel Sewage-Associated Marker Genes in Human and Nonhuman Fecal Samples. Appl. Environ. Microbiol..

[46] Bernhard A.E., Field K.G. (2000). A PCR Assay To Discriminate Human and Ruminant Feces on the Basis of Host Differences in *Bacteroides-Prevotella* Genes Encoding 16S rRNA. Appl. Environ. Microbiol..

[47] Sabino R., Veríssimo C., Cunha M.A., Wergikoski B., Ferreira F.C., Rodrigues R., Parada H., Falcão L., Rosado L., Pinheiro C. (2011). Pathogenic fungi: An unacknowledged risk at coastal resorts? New insights on microbiological sand quality in Portugal. Mar. Pollut. Bull..

[48] Google Earth, ©2019 CNES/Airbus. https://earth.google.com/web/search/Zona+Balnear+da+Prainha,+Azores/@38.6534365,-27.21972419,6.49766264a,418.27412965d,35y,360h,0t,0r/data=CigiJgokCesEdVva90VAEZVuSXJ-8j1AGSM6s_f9kRjAIdK8ln2W6UjA.

[49] Simbiente Açores—Engenharia e Gestão Ambiental (2021). Plano de Gestão da Região Hidrográfica dos Açores 2022–2027—Relatório Técnico: Volume 3. Terceira—Caracterização e Diagnóstico da Situação de Referência.

[50] Holcomb D.A., Knee J., Sumner T., Adriano Z., de Bruijn E., Nalá R., Cumming O., Brown J., Stewart J.R. (2020). Human fecal contamination of water, soil, and surfaces in households sharing poor-quality sanitation facilities in Maputo, Mozambique. Int. J. Hyg. Environ. Health.

[51] Green H.C., Haugland R.A., Varma M., Millen H.T., Borchardt M.A., Field K.G., Walters W.A., Knight R., Sivaganesan M., Kelty C.A. (2014). Improved HF183 Quantitative Real-Time PCR Assay for Characterization of Human Fecal Pollution in Ambient Surface Water Samples. Appl. Environ. Microbiol..

[52] Lu J., Domingo J.W.S., Lamendella R., Edge T., Hill S. (2008). Phylogenetic Diversity and Molecular Detection of Bacteria in Gull Feces. Appl. Environ. Microbiol..

[53] Shanks O.C., Atikovic E., Blackwood A.D., Lu J., Noble R.T., Domingo J.S., Seifring S., Sivaganesan M., Haugland R.A. (2008). Quantitative PCR for Detection and Enumeration of Genetic Markers of Bovine Fecal Pollution. Appl. Environ. Microbiol..

[54] Teixeira P., Dias D., Costa S., Brown B., Silva S., Valério E. (2020). *Bacteroides* spp. and traditional fecal indicator bacteria in water quality assessment—An integrated approach for hydric resources management in urban centers. J. Environ. Manag..

[55] Whitman R.L., Harwood V.J., Edge T.A., Nevers M.B., Byappanahalli M.N., Vijayavel K., Brandão J., Sadowsky M.J., Alm E.W., Crowe A. (2014). Microbes in beach sands: Integrating environment, ecology and public health. Rev. Environ. Sci. Bio. Technol..

[56] Heaney C.D., Sams E., Dufour A.P., Brenner K.P., Haugland R.A., Chern E., Wing S., Marshall S., Love D.C., Serre M. (2012). Fecal Indicators in Sand, Sand Contact, and Risk of Enteric Illness Among Beachgoers. Epidemiology.

[57] Kirs M., Harwood V., Fidler A., Gillespie P., Fyfe W., Blackwood A., Cornelisen C. (2011). Source tracking faecal contamination in an urbanised and a rural waterway in the Nelson-Tasman region, New Zealand. N. Z. J. Mar. Freshw. Res..

[58] Somnark P., Chyerochana N., Kongprajug A., Mongkolsuk S., Sirikanchana K. (2018). PCR data and comparative performance of Bacteroidales microbial source tracking genetic markers. Data Brief.

[59] Zhang Y., Wu R., Lin K., Wang Y., Lu J. (2020). Performance of host-associated genetic markers for microbial source tracking in China. Water Res..

[60] Rinninella E., Raoul P., Cintoni M., Franceschi F., Miggiano G.A.D., Gasbarrini A., Mele M.C. (2019). What Is the Healthy Gut Microbiota Composition? A Changing Ecosystem across Age, Environment, Diet, and Diseases. Microorganisms.

[61] Ashbolt N., Grabow W., Snozzi M., Fewtrell L., Bartram J. (2001). Indicators of microbial water quality. Water Quality: Guidelines, Standards and Health: Assessment of Risk and Risk Management for Water-Related Infectious Disease.

[62] Bolinger H. (2014). The Survival of *Enterococcus faecalis* and *Bacteroides fragilis* on Four Different Food Contact Surfaces. Master’s Thesis.

[63] Elmanama A.A., Fahd M.I., Afifi S., Abdallah S., Bahr S. (2005). Microbiological beach sand quality in Gaza Strip in comparison to seawater quality. Environ. Res..

[64] Halliday E., Gast R.J. (2011). Bacteria in Beach Sands: An Emerging Challenge in Protecting Coastal Water Quality and Bather Health. Environ. Sci. Technol..

[65] Pereira E., Figueira C., Aguiar N., Vasconcelos R., Vasconcelos S., Calado G., Brandão J., Prada S. (2013). Microbiological and mycological beach sand quality in a volcanic environment: Madeira archipelago, Portugal. Sci. Total Environ..

