# Supplementary material for: Microbial Source Tracking as a Method of Determination of Beach Sand Contamination
Source: Int J Environ Res Public Health. 2022 Jun 28;19(13):7934. doi: 10.3390/ijerph19137934 (PMC9265816; doi:10.3390/ijerph19137934)
Supplement: Supplementary file 1 [file ijerph-19-07934-s001.zip › ijerph-1752784-supplementary.pdf]

Protocol for MST analysis of sand as designed for the current study:

a) Sampling: Aseptically collect hand-grab size sand samples into sterile containers, at a depth of 10 cm from the surface, from 3 per area selected (based on pre-screening result (Perform macroscopic observations, divide the area of interest (into logical sections, either due to natural barriers or the obvious signs of contamination - but do not limit the analysis to such a spot) and perform MST using mutiple composite samples (mixed samples from several sites in an area) to obtain general info about the contamination of the area of interest. This may provide unexpected results, and new "areas of contamination" where further analysis may need to be established.)) and transport refrigerated to the laboratory to process within 24h. Mix thoroughly before processing to ensure homogeneity of the sample. Process straight away or freeze the sand samples to be analyzed later on. If mapping sections of the beach is intended, instead of sampling to represent the entire beach, sample individual sections of the beach and process them as unique samples instead of building a composite sample of the entire beach.

b) DNA Extraction usingDNeasy PowerWater Kit (*Qiagen*) with some adaptations: Heat the Solution PW1 to 55°C, for 5 to 10 min, to dissolve any precipitates. In the PowerWater DNA Bead Tube (provided by the Kit), place a small amount of the sand sample (Note: The amount of sand administered in each tube depends on the grain size of the sand. When the sand grain size has a fine grit (≤ 2 mm) place 1.5 g of sand in the tube, whereas when the sand grain size has an intermediate gauge ($2 < x \leq 5$ mm) place 6.0 g in the tube). Add 1 mL of Solution PW1 to the PowerWater DNA Bead Tube. Vortex at maximum speed for 10 min. Transfer the supernatant to a clean 2 mL collection tube (provided by the kit). Centrifuge at 13,000 × g for 1 min at room temperature. Avoiding the pellet, transfer the new supernatant to a clean 2 mL collection tube (provided by the kit). Add 200 μL of Solution IRS and vortex briefly to mix. Incubate in refrigerator (2°C to 8°C) for 5 min. Centrifuge the tubes at 13,000 × g for 1 min. Avoiding the pellet, transfer the supernatant to a clean 2 mL collection tube (provided by the kit). Add 650 μL of Solution PW3 and vortex briefly to mix. Load 650 μL of supernatant onto an MB Spin Column (provided by the kit). Centrifuge at 13,000 × g for 1 min, then discard the flow-through. Repeat until all the supernatant has been processed. Place the MB Spin Column Filter into a clean 2 mL collection tube (provided by the Kit). Add 650 μL of Solution PW4 (shake before use). Centrifuge at 13,000 × g for 1 min. Discard the flow-through and add 650 μL of ethanol (provided by the kit). Centrifuge at 13,000 × g for 1 min. Discard the flow-through and centrifuge again at 13,000 × g for 2 min. Place the MB Spin Column into a clean 2 mL collection tube (provided by the kit). Add 100 μL of Solution EB (Elution Buffer) to the center of the filtration membrane. Centrifuge at 13,000 × g for 1 min. Discard the MB Spin Column. Conserve the extracted DNA (at 4ºC or -20ºC, depending on the brevity to be used), which is ready for future applications.

c) PCR conditions

Analyze the DNA obtained from the fresh or frozen sand samples with the primers indicated in Table 1 by conventional PCR. Use PCR reactions of 25 μL PCR composed of 1x PCR buffer (BIOTAQ DNA polymerase), 1 U of Taq polymerase (BIOTAQ DNA polymerase), 3 mM of $MgCl_2$, 1 mM of dNTPs, 1 μM of each primer pair, and 20-30 μg of DNA template.

PCR negative controls, on which no sample DNA was added, must be included in each set of PCR reactions to monitor contamination. Simultaneously, positive controls (DNA extracted from fecal samples) should also be incorporated.

PCR cycling conditions are as follows: initial denaturation at 95°C for 5 min; 40 cycles of 1) denaturation step at 94°C for 45 s, 2) annealing step for each primers pairs, at temperatures indicated in Table 1 for 45 s, and 3) elongation step at 72°C for 1 min; and a final extension at 72°C for 5 min. Visualize PCR products in 1% agarose gels and using DNA ladder. Consider the fecal contamination from the biological source that produces amplification. For example, if using primers for seagulls, dogs, humans and cows and amplification occurs only with dog primers, that means that the biological source of the fecal contamination is dogs. Consult WHO 2021 for further information on Microbial Source Tracking method [8].

[8] World Health Organization. *Guidelines on recreational water quality: Volume 1 coastal and fresh waters*; World Health Organization: Geneva, Switzerland, 2021; pp. 1-164.
